# Colorimetric 3D microPAD for Multiplexed Detection of Paracetamol and Aspirin in Urine and Saliva

**DOI:** 10.3390/s25061756

**Published:** 2025-03-12

**Authors:** Alberto Abalde-Pujales, Vanesa Romero, Isela Lavilla, Carlos Bendicho

**Affiliations:** Centro de Investigación Mariña, Universidade de Vigo, Departamento de Química Analítica y Alimentaria, Grupo QA2, 36310 Vigo, Spain; alberto.abalde.pujales@uvigo.gal (A.A.-P.); isela@uvigo.gal (I.L.)

**Keywords:** 3D microPAD, paracetamol, aspirin, colorimetric chemosensor, urine, saliva, smartphone detection

## Abstract

**Highlights:**

- Novel colorimetric 3D microPAD for multiplex analysis of paracetamol and aspirin
- 3D microPAD chemosensor combined with digitization using a smartphone camera
- Rapid, sensitive and accurate determination of drugs in urine and saliva
- Potential application as a point-of-care testing for in situ drug screening

**Abstract:**

In this work, a novel 3D μPAD cellulose-based colorimetric chemosensor for multiplexed detection of paracetamol and aspirin in biological samples is proposed. The easy availability of analgesics such as paracetamol and non-steroidal anti-inflammatory drugs such as aspirin, over-the-counter drugs that can be acquired without medical prescription, can entail a health problem if they are administered incorrectly. The development of analytical procedures for the rapid, sensitive, and accurate determination of such drugs in clinical samples is of utmost importance. Different parameters involved in the design of the 3D μPAD system and the colorimetric reaction conditions have been optimized. Under optimal conditions, detection limits of 0.004 mM and 0.013 mM were obtained for paracetamol and aspirin, respectively. The proposed procedure was validated against two certified reference materials and applied to the analysis of several synthetic urine and saliva samples. Synthetic urine and saliva samples were spiked at two concentration levels, showing recoveries in the range of 98–103% with a relative standard deviation of 3–6% (*n* = 6).

## 1. Introduction

The global pharmaceutical market was valued at $943 billion in 2020 and is estimated to grow by 6% through 2026 [1]. Key factors driving the pharmaceutical industry include the increasing prevalence of chronic diseases requiring pharmacological treatment, as well as decreasing regulatory barriers for new drugs. Among the different analgesic and anti-inflammatory drugs, the most recurrent are paracetamol (also known as acetaminophen) and aspirin (i.e., acetylsalicylic acid) [2]. The widespread use of these drugs and their easy availability have positioned them as potentially prone to overdose due to incorrect self-medication or less commonly due to deliberate self-poisoning [3]. The most usual symptoms of paracetamol acute intoxication may include nausea, vomiting, abdominal pain, confusion, and jaundice [4]. In the case of aspirin, the first symptoms of acute intoxication may include ringing in the ears and hearing problems, hyperventilation, vomiting, dehydration, or fainting, and may cause coma in severe cases. The U.S. Food and Drug Administration (FDA) established a maximum recommended dose of 4000 mg/day for paracetamol and aspirin. In addition, it is recommended to limit the maximum dose per intake of paracetamol and aspirin in adults to a maximum of 650 mg [4]. Despite the recommendations, self-intoxications with paracetamol and aspirin have increased in the last few years by 33.5% among children (6 to 12 years) and adolescents (13 to 19 years), reaching its peak between 2019 and 2021, a period that coincided with the COVID-19 pandemic [3]. The easy availability of analgesics and anti-inflammatory drugs that could be available without medical prescription raises concerns about their misuse.

The determination of paracetamol and aspirin in human clinical samples, such as blood, urine, and saliva, usually involves the use of chromatographic techniques [5,6,7,8,9,10,11,12]. Within the group of chromatographic methods, most of the reported procedures are based on liquid chromatographic (LC) coupled to mass spectrometry (MS) and diode array detector (DAD) for quantification. Although chromatographic methods combined with adequate sample pretreatment offer the advantage of being able to simultaneously determine paracetamol and aspirin and their metabolites at low concentration levels, it should also be noted that these procedures entail the use of centralized equipment usually involving time-consuming protocols and high consumption of organic solvents.

An advance in the simplification of the procedure for the rapid detection of paracetamol and aspirin in clinical samples would be the implementation of point-of-care testing (PoCT) devices, whose characteristics are close to the ASSURED (affordable, sensitive, specific, user-friendly, rapid and robust, equipment-free, and deliverable to end-users) concept [13]. Thus, avoiding the use of centralized instrumentation or tedious procedures and reducing cost and time. In this context, paper-based microfluidic analytical devices (μPADs) have emerged as promising tools due to their low cost, low sample consumption, easy fabrication, and easy use [14]. Different electrochemical paper-based analytical devices (ePADs) have been reported for the analysis of paracetamol in commercial pharmaceutical products [15], gin [16], wastewater [17], and urine [18]. Besides, recently, W.K.T. Coltro et al. [19] and A. Hussen et al. [20] have developed colorimetric approaches based on two-dimensional (2D) μPADs for the determination of paracetamol in whisky and water, offering simpler preparation strategies in comparison to ePADs. In the case of aspirin, R. Jain et al. [21] recently reported a procedure based on the combination of vortex-assisted dispersive liquid–liquid microextraction with colorimetric detection on a filter paper impregnated with a chromogenic reagent following smartphone-based digitalization for quantification.

It should be noted that the reported works are focused on the individual analysis of paracetamol or aspirin. Furthermore, colorimetric μPAD systems have not been applied for the detection of paracetamol in clinical samples, such as saliva and urine. In the case of application of colorimetric μPADs to clinical samples, it is very interesting to assess multiplexed analysis allowing the simultaneous determination of different analytes at the same time. Thus, 3D μPAD systems add a vertical dimension to the horizontal directions of 2D μPADs offering multiplexed detection and multistage analytical processes [22]. With this premise, in this work, a new method based on a 3D μPAD cellulose-based colorimetric chemosensor for multiplexed detection of paracetamol and aspirin in biological samples is proposed. Dry reagents supported on the cellulose substrates are applied for generating colored products, which are easily digitized by electronic devices such as a smartphone camera and subsequently processed to obtain the quantification of the analyte. The application of the novel approach to the determination of paracetamol and aspirin in different synthetic urine and saliva samples is demonstrated.

## 2. Materials and Methods

### 2.1. Reagents and Solvents

A stock standard solution (0.66 mM) of paracetamol was prepared by dissolving the required amount of commercial paracetamol (98%, Acros Organics, Antwerp, Belgium) in ultrapure water. A stock standard solution (4.44 mM) of aspirin was prepared by dissolving the commercial acetylsalicylic acid (99.5%, Panreac, Barcelona, Spain) in 1 M NaOH. Diluted working aqueous standards were prepared fresh daily from the stock solutions by dilution with ultrapure water. For the colorimetric reactions, FeCl_3_ (98.8%, Sigma-Aldrich, Steinheim, Germany), K_3_[Fe(CN)_6_] (Probus, Badalona, Spain), and NaOH (99.1%, Prolabo, Fontenay-sous-Bois, France) were used.

For the synthesis of synthetic urine and saliva samples, the following reagents were employed: K_2_HPO_4_ (Panreac, Barcelona, Spain), NaHCO_3_ (Carlo Erba, Milán, Italy), NaCl (Sigma-Aldrich, Steinheim, Germany), MgCl_2_ (99%, Prolabo, Fontenay-sous-Bois, France), C_6_H_8_O_7_ (99.5–102%, Sigma-Aldrich, Steinheim, Germany), CaCl_2_ (94%, Prolabo, Fontenay-sous-Bois, France), CH_4_N_2_O (99%, Sigma-Aldrich, Steinheim, Germany), Na_3_C_6_H_5_O_7_ (99%, Sigma-Aldrich, Steinheim, Germany), Na_2_SO_4_ (99%, Sigma-Aldrich, Steinheim, Germany), NH_4_Cl (99.9%, Sigma-Aldrich, Steinheim, Germany), KCl (99%, Prolabo, Fontenay-sous-Bois, France), C_5_H_4_N_4_O_3_ (99%, Sigma-Aldrich, Steinheim, Germany), C_4_H_7_N_3_O (≥98%, Sigma-Aldrich, Steinheim, Germany), K_2_C_2_O_4_ (≥98.5%, Sigma-Aldrich, Steinheim, Germany), and MgSO_4_ (99.9%, Sigma-Aldrich, Steinheim, Germany).

All reagents used were of analytical grade or higher. The ultrapure water was obtained from a Simplicity^®^ water purification system from Merck Millipore with a resistivity of 18.2 MΩ·cm (Darmstadt, Germany).

### 2.2. Instrumentation and Materials

A Galaxy S8 smartphone from Samsung (Seoul, Republic of Korea) and a portable PULUZ photo studio lightbox (PULUZ Technology Limited, Shenzhen, China) equipped with 20 LEDs were used for smartphone-based colorimetric detection. Furthermore, a Galaxy A53 smartphone from Samsung (Seoul, Republic of Korea) and a 11T smartphone from Xiaomi (Beijing, China) were used for the comparison of results between different devices. A ColorQube 8580 printer from Xerox (Rochester, New York, NY, USA) and a drying oven, model 2001244 from P-selecta (1200 W, 50/60 Hz) (Barcelona, Spain) were used for defining hydrophobic barriers on cellulose filter paper No. 541 from Whatman (Maidstone, Kent, UK) by wax printing technology.

### 2.3. Data Processing

The App RGB Color Detector (The Programmer, Google Play Store) and Color Grab (Loomatix, Google Play Store) were used for data acquisition through the smartphone camera. Additionally, the free image processing program ImageJ version 1.54g and the free web application Trigit (accessible at: https://trigit.com.au/) were used for this purpose.

### 2.4. Design and Fabrication of 3D μPADs

**Figure 1:**
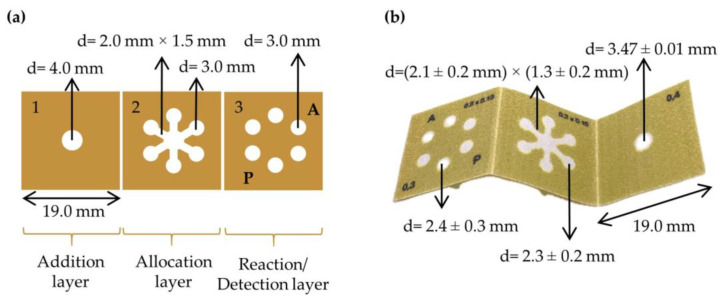
Schematic design of 3D μPAD. Computer scheme (**a**); printed and folded 3D μPAD after heating (**b**). Reaction/detection reservoirs marked as P are for paracetamol detection, reaction/detection reservoirs marked as A are for aspirin detection.

3D μPADs were designed by creating hydrophobic ink patterns printed on hydrophilic cellulose substrates using a wax printer. The 3D μPAD design is shown in Figure 1. It consists of three square layers: addition layer (1), allocation layer (2), and reaction/detection layer (3) (19.0 mm × 19.0 mm each). First, layer (1) includes a circular shaped (d = 4.0 mm) hydrophilic area, used as the inlet for blanks, standards, or samples. Second, layer (2) includes a circular shaped (d = 4.0 mm) hydrophilic area aligned in position with the hydrophilic part of layer (1) and six circular hydrophilic areas (d = 3.0 mm) connected to the central zone by hydrophilic channels (2.0 mm long and 1.5 mm wide). Finally, layer (3) has six circular hydrophilic areas (d = 3.0 mm) aligned in position with layer (2). On this last layer, the dry reagents for colorimetric determination are pre-deposited.

For the fabrication of the 3D μPAD, Whatman No. 541 filter paper was used as a hydrophilic substrate. Prior to printing the wax pattern, the cellulose substrate is heated at 130 °C for 2 min. The wax pattern is then printed on the cellulose substrate and heated again at 130 °C for 40 s to embed the hydrophobic wax into the cellulose fiber matrix. Each 3D μPAD was cut manually prior to use.

### 2.5. Experimental Procedure for the Determination of Aspirin and Paracetamol

First, on layer (3) of the unfolded 3D μPAD (Figure 1b), 5 μL of 7.5 mM [K_3_Fe(CN)_6_]:FeCl_3_ at equimolar concentration were deposited on the three reaction/detection reservoirs marked as P (for paracetamol detection), and 5 μL of 20 mM FeCl_3_ were deposited on the three detection reservoirs marked as A (for aspirin detection)**.** Subsequently, the 3D µPAD was heated in the oven for 5 min at 60 °C, thus obtaining the detection reservoirs with the dried reagents. The dried reagents were deposited on the 3D μPAD daily, ensuring optimal performance and analytical reliability.

**Figure A1:**
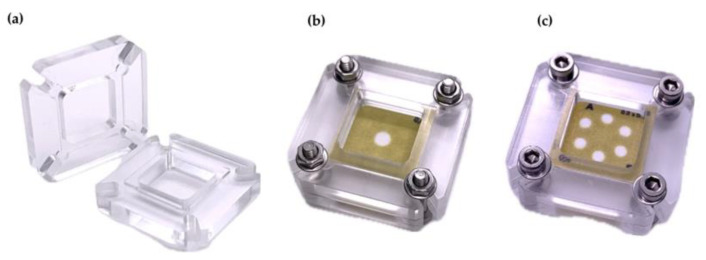
Methacrylate support: empty (**a**); with 3D μPAD showing the addition layer (**b**) and the reaction/detection layer (**c**).

The 3D μPAD was then folded and placed on a methacrylate support (Figure A1) [22]. Then, 22 μL of aqueous solution (blank, standard, or sample) was deposited in the addition area of layer (1). Then, the methacrylate support was rotated, leaving the detection zone visible, and placed inside a light booth for photography with controlled luminosity. After 2 min of reaction, the color generated in the detection zones was digitized by the smartphone camera (ISO = 200, EV = +2, WB = 5000 K for paracetamol and ISO = 50, EV = +1, WB = 5000 K for aspirin). The analytical response (mean color intensity difference in the red channel for paracetamol and green channel for aspirin, ΔIc = blank − standard) of each detection area was obtained by processing the image using RGB Color Detector app version 3.0.91 or ImageJ software version 1.54g.

### 2.6. Preparation of Synthetic Saliva and Urine Samples

The application of this method includes the analysis of four synthetic clinical samples, including saliva and urine, which were prepared as follows. Synthetic saliva (1) was prepared in accordance with the reference method [23]. For synthetic saliva (2), the reference method [24] was used. For synthetic urine (1), the reference method [25] was used, and finally, for synthetic urine (2), the reference method [26] was used. Both synthetic saliva and urine samples were stirred for 15 min. Detailed procedures for synthetic sample preparation are described in the Supplementary Material (SM).

### 2.7. Fundamentals of the Colorimetric Reactions

On the one hand, the paracetamol reaction is based on an adaptation of the methodology first used by S. Abed [27] and later adapted by F. Pourkarim [28]. In this case, the paracetamol reduces Fe(III) from potassium ferricyanide to Fe(II), forming potassium ferrocyanide, which subsequently reacts with FeCl_3_ to form the characteristic Prussian Blue complex [19].

On the other hand, aspirin reacts with FeCl_3_, producing a characteristic color change from colorless to violet or deep purple, depending on the analyte concentration. Aspirin in a basic medium forms the salicylate dianion. The salicylate dianion reacts with FeCl_3_, where Fe(III) coordinates with the phenolic group and the carboxylate group of the salicylate anion, forming the characteristic-colored complex, the tetraacuosalicylate-Fe(III) ion, FeSA [21,29].

## 3. Results and Discussion

### 3.1. Optimization of Experimental Parameters

Experimental variables associated with the design of the 3D μPAD, digitization, image processing conditions, as well as other parameters affecting the formation of the colored products in the detection areas for the determination of paracetamol and aspirin, were optimized. Experiments were carried out using standards of 0.07 mM paracetamol and 2.50 mM aspirin.

#### 3.1.1. Selection of the Study Channel

**Figure 2:**
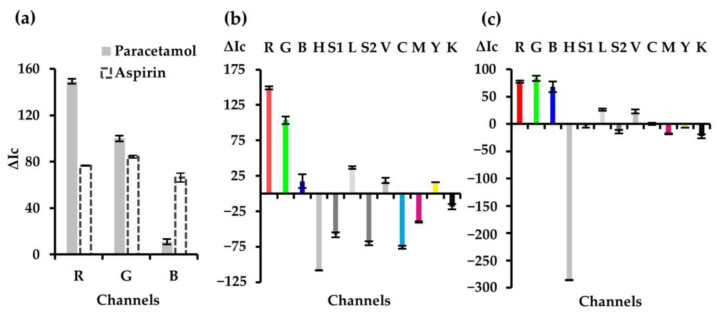
Effect of RGB color channels on the analytical response (**a**). Effect of CMYK, HSL, and HSV color channels on the analytical response for the colorimetric determination of paracetamol (**b**) and aspirin (**c**).

The selection of the most appropriate RGB color channel was first performed. For this purpose, the experiments were performed both in the absence (blank) and presence of the target analytes. As shown in Figure 2a, the R channel provides the maximum analytical response for paracetamol and the G channel for aspirin. These results were expected since, for paracetamol, an absorption band at around 748 nm (red range) was obtained due to the blue product formed. In the case of aspirin, an absorption band at 522 nm (green range) was obtained due to the purple/pink complex formed. In addition to confirming this, the rest of the channels (CMYK, HSL, HSV) shown in Figure 2b,c were also tested.

#### 3.1.2. Drying Temperature of the Colorimetric Reagents

**Figure 3:**
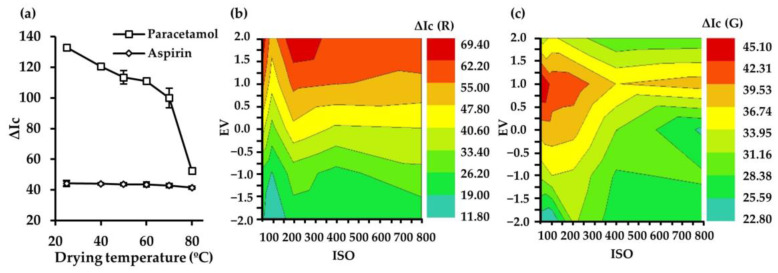
Effect of drying temperature of the colorimetric reagents on the analytical response (**a**). Effect of digitization parameters (ISO and EV) on the analytical response for the colorimetric determination of paracetamol (**b**) and aspirin (**c**).

The aim of this study was to evaluate the reaction stability and optimize the drying temperature of the colorimetric reagents deposited on the cellulose substrate. Therefore, temperatures in the range of 25–80 °C were tested. As shown in Figure 3a, a negative effect on the analytical response was observed as the temperature increased for paracetamol. In the case of aspirin, the analytical response did not vary significantly in the tested range. Thus, a drying temperature of 25 °C was selected as a compromise for both analytes.

#### 3.1.3. Digitalization and Processing Conditions

**Figure 4:**
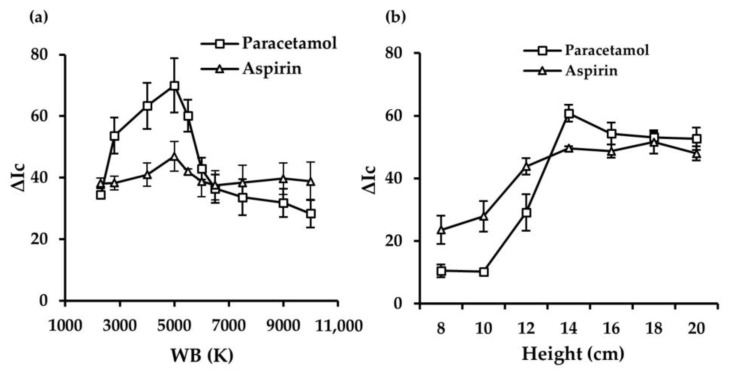
Effect of WB values for paracetamol and aspirin with ISO and EV fixed (**a**). Optimization of image acquisition height for paracetamol and aspirin with ISO, EV, and WB fixed (**b**).

It is crucial to establish optimal digitization conditions to achieve maximum sensitivity for paracetamol and aspirin detection. The effect of three photographic parameters, including the International Organization for Standardization (ISO), exposure value (EV), and white balance (WB), were evaluated. EV combines shutter and aperture speed, whereas ISO indicates the camera’s sensitivity and controls the amount of light it lets through [30]. In addition, WB adjusts the color temperature of the image, i.e., the warm or cold hue of the recorded image. As can be observed in Figure 3b,c, the analytical response significantly increased on increasing both ISO and EV, showing the highest analytical response when the ISO and EV were set at 200 and +2.0 for paracetamol, respectively. For aspirin, the optimal conditions were set at ISO = 50 and EV = +1.0. These digitization conditions were, therefore, selected for subsequent studies. Smartphone camera settings allowed the selection of WB in a range between 2300 and 10,000 K. Results shown in Figure 4a indicate that for both analytes, the maximum signal was reached with a WB value of 5000 K, which was selected for the following studies for the following studies.

Furthermore, the distance between the smartphone camera and the 3D μPAD was tested [31]. The distance between the 3D μPAD and the smartphone camera during the acquisition of the image was related to the incidence of light on the PAD. The camera was placed at an angle of 0° with respect to the position of the 3D μPAD placed in the methacrylate support, varying only the distance between the two components. The distance varied between 8 and 20 cm, leaving the PAD in a fixed position and placing the camera at different heights inside the light booth for photography. The angle at which the image was acquired did not vary, since it could significantly modify the color response obtained in the image processing [32]. As shown in Figure 4b, at distances less than 14 cm, the analytical signal was lower since the camera was below the minimum focusing distance, and, therefore, the image recorded is blurred, affecting the processing. Distances greater than 20 cm imply placing the camera in the external area of the light booth for image acquisition, and thus the image was affected by the external light. Thus, distances > 20 cm were discarded. Finally, an image acquisition height of 14 cm was selected to obtain the highest analytical response.

#### 3.1.4. Type of Cellulose Substrate

**Figure 5:**
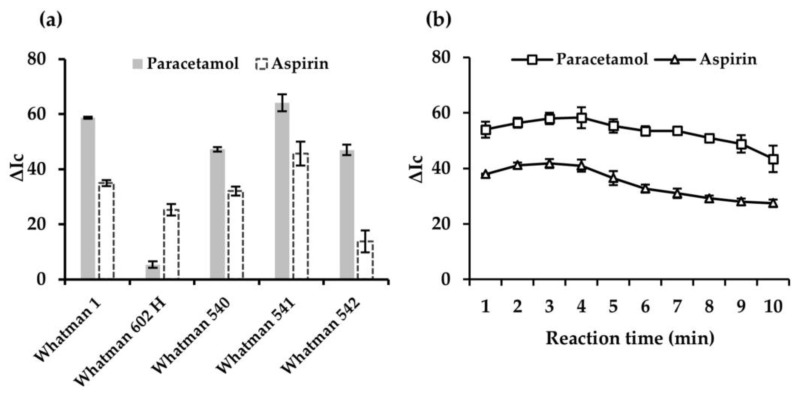
Effect of the type of cellulose substrate on the analytical response (**a**). Effect of digitization time on the analytical response (**b**).

The analytical performance of five cellulose substrates, namely Whatman No. 1, 602H, 540, 541, and 542, with different particle sizes, particle retention, and chemical treatment was evaluated. As shown in Figure 5a, the best results were obtained with Whatman 541. This could be due to two key parameters in the type of paper, such as its pore size and thickness. In reference to the first one, considering that the PAD used is a 3D system, where the aqueous aliquot needs to circulate by capillarity through the different layers of the cellulose substrate, a large pore size may allow a faster diffusion improving the uniformity of the color generated in the detection zone of the substrate. On the other hand, the thickness of the substrate can affect the color intensity generated in the detection zone, since the greater the thickness, the smaller the color gradient observed. In this sense, dry reagents are distributed over a greater thickness of the opaque cellulose substrate, reducing the color intensity recorded in the surface zone.

#### 3.1.5. Effect of the Digitation Time

To establish the optimum digitization time, i.e., time elapsed from the deposition of the blank, standard, or sample is dropped on the addition layer until the images are captured, a study of the color intensity generated in the detection zone versus time was performed. This parameter is important to ensure maximum sensitivity with an adequate sampling frequency. Different digitizing times were tested in the interval of 2–10 min. As shown in Figure 5b, as the time increased, the signal obtained increased until reaching a plateau in the interval of 2–4 min. This time interval is appropriate in order to design a 3D μPAD prototype that includes the two drugs simultaneously, since an optimal time window was obtained to first digitize the aspirin detection areas with its conditions (ISO = 50, EV = +1.0 and WB = 5000 K) and then paracetamol (ISO = 200, EV = +2.0 and WB = 5000 K).

#### 3.1.6. Design of the 3D μPAD: Reservoir and Channel Sizes

**Figure 6:**
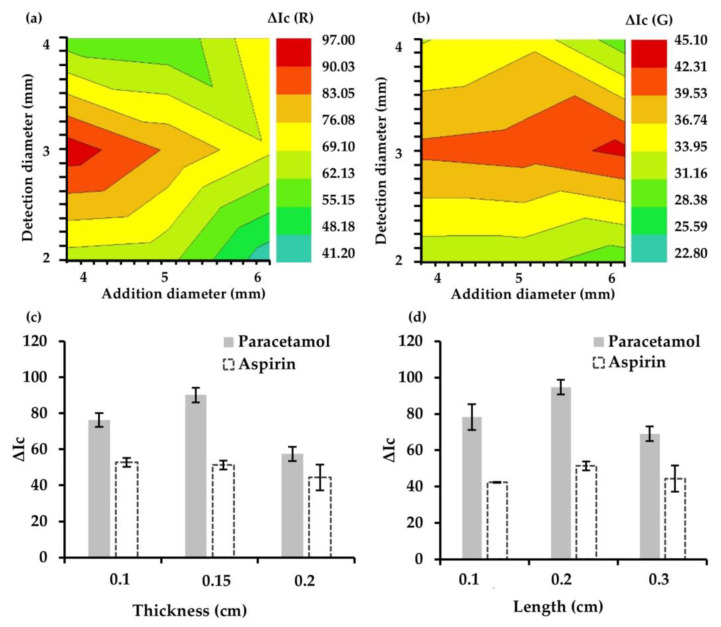
Effect of the diameter of addition and detection layers of μPAD on the analytical response for the colorimetric determination of paracetamol (**a**) and aspirin (**b**). Optimization of thickness (**c**) and length (**d**) of the hydrophilic channels in the allocation layer.

The size of the reservoirs in the three layers of the 3D μPAD, namely addition, allocation, and reaction/detection layers, is of utmost importance. To optimize these parameters, different 3D μPADs were tested with addition reservoir diameters between 4 and 6 mm and reaction/detection reservoir diameters between 2 and 4 mm. As illustrated in Figure 6a,b, the design of the 3D μPAD significantly influenced both sensitivity and repeatability, due to the varying uniformity of the colored products in the detection zone. Diameters of 4 mm for the addition reservoirs and 3 mm for the detection reservoir were chosen to achieve the best sensitivity and precision. Smaller diameters for the detection reservoirs may interfere with image digitizing because some of the concentrated dry reagents may be retained in the contour of the detection zone. In addition, larger diameters would hinder the color intensity and improper homogenization of the detection layer was observed. Excessively large diameters for the detection reservoirs can also impair the design of a multiplexing feature with several properly spaced hydrophilic areas.

Furthermore, optimization of the thickness and length of the hydrophilic channels is necessary to control the transport of liquids from the addition reservoir to the reaction/detection reservoir. First, the hydrophilic channel thickness was optimized by performing studies in the range of 1–2 mm. It should be noted that the design of the channel thickness prior to thermal treatment in the oven has slightly larger dimensions, as the channels become narrower when the wax is heated. Narrow hydrophilic channels will have less radial diffusion, resulting in less dilution of the color on the hydrophilic substrate. Then, a channel thickness of 1.5 mm was fixed, achieving a correct capillarity and fast absorption of liquids. For sizes smaller than 1.5 mm, it was observed that the wax, once heated, completely closed the channels, thus preventing the correct function of the microfluidic channel.

Finally, the length of the microfluidic channels placed in the allocation layer (layer 3) of the 3D μPAD was optimized by designing paths with different lengths between 1 and 2 mm. In addition, this range was set to use the PAD space as much as possible in order to design the optimal multiplex device. As a compromise between fast transport, which is provided by short distances, and preventing dried reagents from diffusing toward the central layer of the 3D μPAD, a length of 2 mm was set as the optimum value. It allows the maximum sensitivity of the PAD with the highest analytical response (Figure 6c,d).

#### 3.1.7. Effect of Sample Volume

**Figure 7:**
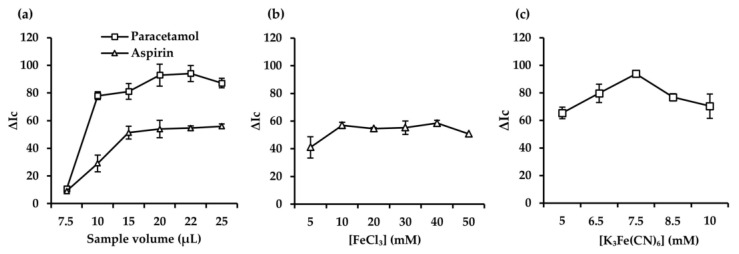
Effect of sample volume on the analytical response (**a**). Optimization of FeCl_3_ concentration (**b**). Optimization of [K_3_Fe(CN)_6_]:FeCl_3_ concentration (**c**).

The color intensity of the products formed in the detection zone is directly related to the sample volume. The effect of sample volume was studied in the range of 7.5–25 µL (Figure 7a). An increase in the analytical response was observed as the sample volume increased until a maximum was reached for a sample volume of 22 µL for both analytes. This can be attributed to the fact that a volume of 22 µL was sufficient to reliably fill all six detection zones. Also, lower sample volumes led to incomplete wetting of the detection zones, worsening the analytical response. Volumes above 25 µL were discarded for the designed 3D μPAD since they exceeded the surface tension of the droplet, causing its transfer to the hydrophobic zone by saturating the cellulose.

#### 3.1.8. Effect of FeCl_3_ Concentration

The added amount of FeCl_3_ in the reaction medium plays an important role in aspirin detection, as it directly influences the color intensity signal and the process of complex formation with the resulting analytical response [29]. To study the effect of this parameter, 5 µL of FeCl_3_ solution at different concentrations (i.e., 5–50 mM) was deposited in the detection reservoir of the 3D µPAD. As shown in Figure 7b, the analytical response (ΔIc) increased until reaching a maximum at FeCl_3_ 20 mM and remained constant up to a concentration of 40 mM. For higher FeCl_3_ concentrations, a decrease in the analytical response was observed, which can be attributed to the formation of a precipitate on the cellulosic substrate, hence preventing the correct determination of the target analyte.

#### 3.1.9. Effect of K_3_[Fe(CN)_6_] Concentration

The amount of [K_3_Fe(CN)_6_]:FeCl_3_ mixture directly influences paracetamol detection. The reduction process of Fe(III) to Fe(II), where paracetamol acts as a reducing agent for the subsequent formation of the Prussian Blue complex, is directly related to the concentration of the reagent deposited on the 3D µPAD [33]. To evaluate the influence of this parameter, different concentrations of the mixture [K_3_Fe(CN)_6_]: FeCl_3_ (at equimolar concentration) were tested in the range of 5–10 mM.

As shown in Figure 7c, the analytical response (ΔIc) for paracetamol increased as the concentration of the reagent rised, reaching a maximum at 7.5 mM. For concentrations higher than 7.5 mM, a decrease in the analytical response was observed, which could be attributed to the fact that the reagent mixture exhibits color in the absence of paracetamol. This causes a negative influence on the color generated after the reaction with the target analyte, hence impairing the analytical response and sensitivity. Finally, a concentration of 7.5 mM was selected as the optimal K_3_[Fe(CN)_6_] concentration.

### 3.2. Effect of Interferences

The effect of potential interferences in the determination of paracetamol and aspirin was assessed. Substances were considered to interfere when a variation of more than ±10% of the analytical signal was observed. Table 1 shows the tolerance limits obtained by the proposed method, as well as the reported concentration ranges for the evaluated species. It can be deduced from the data shown in Table 1 that the proposed method shows a relatively high tolerance to common species, far beyond the levels typically found in human saliva and urine samples.

### 3.3. Comparison of Different Smartphones and Different Methods for Paracetamol and Aspirin Quantification

Three distinct smartphone models were tested under optimal digitization conditions (ISO, EV, WB). Furthermore, four different methods were used to analyze the images obtained in order to quantify the target analytes. The obtained results are shown in Table 2. It is observed that the analytical response obtained did not vary when different devices were used to acquire the images. Besides, it was demonstrated that the use of different applications or software for image processing did not lead to a variation in the analytical response. No significant differences were observed between the added and found values when applying a *t*-test (α = 0.05).

### 3.4. Analytical Characteristics

**Figure A2:**
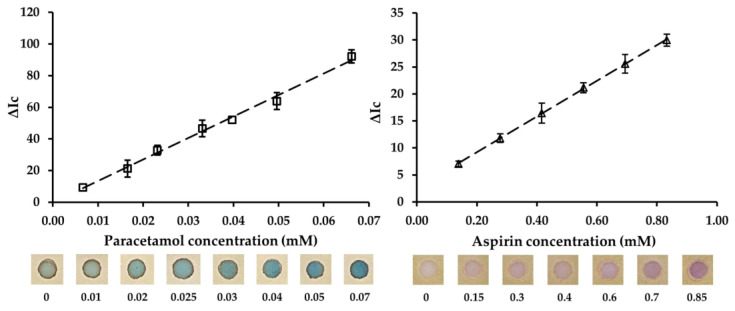
Linear functions of the calibration curve for paracetamol and aspirin along with the original images of the calibration at different concentrations (numbers below images indicates concentration in mM of each analyte).

Analytical characteristics for the proposed method are shown in Table A1. Calibration curves were obtained by plotting the analytical response ΔIc (expressed as blank color intensity − standard/sample color intensity) against paracetamol or aspirin concentration expressed as mM. The linear functions of the calibration curve are shown in Figure A2. Calibration curves were linear, up to 0.07 mM and 0.85 mM for paracetamol and aspirin, respectively. The limits of detection (LODs) calculated according to the 3σ IUPAC criteria were 0.004 mM and 0.013 mM for paracetamol and aspirin, respectively. Besides, the limits of quantification (LOQs) according to 10σ IUPAC criteria were 0.013 mM for paracetamol and 0.045 mM for aspirin. Furthermore, the repeatability and reproducibility (calculated as between-day precision) values, expressed as relative standard deviation (RSD, %) and tested at two concentration levels, were in the range of 3.3–6.1%.

A comparison of the proposed approach with other reported procedures for the determination of paracetamol and aspirin is shown in Table 3. Chromatographic methods stand out for their LOD, allowing the determination of paracetamol and aspirin at low concentration levels. However, it should be pointed out that procedures based on chromatographic methods are usually lengthy with tedious sample pretreatment. Also, ePADs offer high sensitivity toward paracetamol, but, in general, sophisticated electrodes and multistep preparation procedures are needed for fabricating the ePAD, increasing costs and accessibility.

In addition, comparing the proposed methodology to other recent works that implement colorimetric PADs, the LODs obtained with the proposed procedure are comparable in the case of paracetamol and improved those reported for aspirin. As can be observed, one of the main advantages of the proposed procedure is the reduction in the time needed for analysis, which makes it a suitable complement to chromatographic analyses providing an initial screening of samples. This is due to the rapid formation of both complexes on the cellulosic substrate and the one-step combination of direct detection of both analytes. The proposed procedure offers a cost-effective PAD with a total cost of 0.30 € per 3D µPAD. Finally, it should be added that this is the first work where a cellulose substrate is implemented for the determination of both drugs in clinical samples.

### 3.5. Application of the Proposed Method to Clinical Samples

The method was validated against two certified reference materials (CRMs): ANSI National Accreditation Board^®^ Aspirin from Supelco (Darmstadt, Germany) with a certified value of 99.9% ± 0.3% (as mass fraction g/g) and ANSI National Accreditation Board^®^ Acetaminophen from Supelco (Darmstadt, Germany) with a certified value of 99.99% ± 0.04% (as mass fraction g/g). Concentrations found for the CRMs were 102.5% ± 4.2% for paracetamol and 96.6% ± 5.1% for aspirin, which were in good agreement with the certified values. Finally, four clinical samples were analyzed by the proposed method for the determination of paracetamol and aspirin. The selected samples were synthetic urine and synthetic saliva. To evaluate the accuracy of the method, recovery studies were carried out at two concentration levels, i.e., 0.02 mM and 0.05 mM for paracetamol; 0.30 mM and 0.85 mM for aspirin. The obtained results are shown in Table 4. The recoveries obtained were quantitative for the levels of paracetamol and aspirin studied (AOAC, Association of Official Analytical Chemists), showing no significant differences between the certified and found values when applying a *t*-test (α = 0.05).

## 4. Conclusions

The implementation of a colorimetric chemosensor based on a 3D μPAD format has enabled the development of a new analytical procedure for the simultaneous colorimetric determination of paracetamol and aspirin in clinical samples. Image acquisition with a smartphone and its processing on the same electronic device enable the quantification of both analytes in a simple and fast way. Paracetamol is determined through the formation of a Prussian Blue complex, while for aspirin, a purple complex develops. One of the main advantages of the proposed method is the short analysis time, as direct detection is accomplished in less than 4 min. Under optimal conditions, LODs of 0.004 mM and 0.013 mM were obtained for paracetamol and aspirin, respectively. The use of a 3D μPAD allows for a cheap, fast, easy, and simple way for the in situ measurement of paracetamol and aspirin in human biological samples without the need for specialized personnel. This offers the possibility of its application as a point-of-care testing (PoCT) method for the total determination of these drugs in cases of suspected deliberate self-intoxication, which can be combined with centralized analytical techniques such as liquid or gas chromatography for confirmation.

## Figures and Tables

**Table 1:** Effect of potential interferences in urine and saliva on the application of the 3D colorimetric μPAD.

Potential Interference	Studied Range (mM)	Reported Levels in Saliva (mM)	Reported Level in Urine (mM)	Refs.
Na^+^	300	0.002–18.8	0.53–220	[34,35,36,37,38]
K^+^	150	0.010–19.2	3.03–125	[34,35,36,37,38]
Mg^2+^	50	0.001–0.3	0.18–15	[34,35,36,37,38]
Ca^2+^	50	0.002–1.03	0.44–10	[36,37,38,39,40]
Cl^−^	300	0.003–18.8	0.32–250	[34,38,41]
HPO_4_^2−^	50	0.001–0.24	4.82–22.81	[34,41]
HCO_3_^−^	100	1–60	n.a	[42]
Citric acid	50	0.001–0.024	n.a	[40]
NH_4_^+^	100	n.a	0.17–70	[37]
C_2_O_4_^2−^	50	n.a	0.109–0.497	[43,44]
SO_4_^2−^	100	n.a	10–40	[45,46]
Citrate	50	n.a	0.49–2.55	[43,47]
Uric acid	50	n.a	0.42–5.95	[46]
Urea	750	n.a	333–582.75	[46]
Creatinine	50	n.a	5.23–29.2	[43,47]

n.a: Not available.

**Table 2:** Comparison between smartphones and different applications for drug quantification.

App	RGB Color Detector		Image J		Trigit		Color Grab	
Device	Paracetamol	Aspirin	Paracetamol	Aspirin	Paracetamol	Aspirin	Paracetamol	Aspirin
S1 *	100 ± 3	99 ± 6	97 ± 3	97 ± 5	100 ± 3	99 ± 6	102 ± 7	105 ± 3
S2 *	94 ± 4	102 ± 3	94 ± 3	102 ± 4	100 ± 4	99 ± 4	94 ± 4	95 ± 3
S3 *	99 ± 4	95 ± 3	99 ± 4	100 ± 4	96 ± 4	94 ± 5	105 ± 2	98 ± 3

* S1: Samsung Galaxy S8; S2: Samsung Galaxy A53; S3: Xiaomi 11T smartphone.

**Table 3:** Comparison of methods for paracetamol and aspirin determination.

Method	Analyte	Sample	LOD (mM)	RSD (%)	Sample Volume (µL)	Analysis Time (min)	Refs.
*Chromatographic methods*							
HPLC—UV	Paracetamol	Urine	2.2 × 10^−6^	3.54	20	20	[5]
HPLC—DAD	Paracetamol	Synthetic urine and wastewater	21.9 × 10^−6^	2.5	10	13	[6]
HPLC—UV	Aspirin	Saliva	1.8 × 10^−4^	8.51	10	20	[9]
GC—MS	Aspirin	Urine	6.6 × 10^−7^	4.7	1	6	[12]
*Electrochemical paper-based analytical devices (ePADs)*							
ePAD/BCF	Paracetamol	Pharmaceutical products	3.5 × 10^−9^	0.25	150	1	[15]
PoP-PAD	Paracetamol	Gin	0.015	4	40	15	[16]
2D ePAD	Paracetamol	Wastewater	1.5 × 10^−9^	7	50	1	[17]
ePAD-RGO-CuNP/GCE	Paracetamol	Urine	0.024	3.97	100	10	[18]
*Colorimetric paper-based analytical devices (PADs)*							
2D µPAD distance measurements	Paracetamol	Whisky	0.026	6	17	20	[19]
2D µPAD	Paracetamol	Water	2 × 10^−4^	<2	30	10	[20]
VA-DLLME-DIC	Aspirin	Urine	0.049	<10	5	30	[21]
3D µPAD smartphone—colorimetric detection	Paracetamol	Synthetic urine and synthetic saliva	0.004	5.9	20	2–4	This work
	Aspirin		0.013	3.7			

**Table 4:** Recovery studies for paracetamol and aspirin in synthetic urine and saliva.

Sample	Analyte	Spiked Value (mM)	Found Value (mM ± s, *n* = 3)	Recovery (% ± s, *n* = 3)
Synthetic urine—1	Paracetamol	0.02	0.02 ± 0.01	99 ± 3
		0.05	0.05 ± 0.01	99 ± 2
	Aspirin	0.3	0.28 ± 0.02	102 ± 6
		0.85	0.84 ± 0.05	101 ± 6
Synthetic saliva—1	Paracetamol	0.02	0.02 ± 0.01	100 ± 3
		0.05	0.05 ± 0.01	101 ± 4
	Aspirin	0.3	0.28 ± 0.01	99 ± 5
		0.85	0.83 ± 0.03	98 ± 3
Synthetic urine—2	Paracetamol	0.02	0.02 ± 0.01	102 ± 2
		0.05	0.05 ± 0.01	100 ± 3
	Aspirin	0.3	0.30 ± 0.02	100 ± 2
		0.85	0.86 ± 0.03	103 ± 4
Synthetic saliva—2	Paracetamol	0.02	0.02 ± 0.01	102 ± 2
		0.05	0.05 ± 0.01	98 ± 3
	Aspirin	0.3	0.28 ± 0.03	103 ± 4
		0.85	0.82 ± 0.03	101 ± 6

## Data Availability

The original contributions presented in this study are included in the article/Appendix A. The raw data supporting the conclusions of this article will be made available by the authors on request.

## Supplementary Materials

The following supporting information can be downloaded at: https://www.mdpi.com/article/10.3390/s25061756/s1, Section S1: Procedure for synthetic saliva and urine samples.

## Abbreviations

The following abbreviations are used in this manuscript:

2D	two dimensional
3D	three dimensional
AOAC	Association of Official Analytical Chemists
CMYK	cyan, magenta, yellow, key
CRM	certified reference materials
DAD	diode array detector
ePAD	electrochemical paper-based analytical device
ePAD-RGO-CuNP/GCE	glassy carbon electrode modified with reduced graphene oxide doped with copper nanoparticles electrochemical paper analytical device
ePAD/BCF	electrochemical paper-based device modified with functionalized bamboo-derived biochar
EV	exposure value
FDA	Food and Drug Administration
FeSA	tetraacuosalicylate-Fe(III) ion
HPLC	high-performance liquid chromatography
HSL	hue, saturation, lightness
HSV	hue, saturation, value
ISO	International Organization for Standardization
IUPAC	International Union of Pure and Applied Chemistry
LC	liquid chromatographic
LOD	limit of detection
LOQ	limit of quantification
MS	mass spectrometry
PAD	paper-based analytical device
μPAD	microfluidic paper-based analytical device
PoCT	point-of-care testing
PoP-PAD	pen-on-paper paper-based analytical device
RGB	red, green, blue
RSD	relative standard deviation
UV	ultraviolet
VA-DLLME-DIC	vortex-assisted dispersive liquid–liquid microextraction with smartphone-based digital image colorimetry
WB	white balance

## Appendix A

**Table A1:** Analytical figures of merit of the proposed methodology for paracetamol and aspirin determination in clinical samples.

Parameter	Value	
	Paracetamol	Aspirin
Linear range (mM)	LOQ—0.07	LOQ—0.85
LOD (mM)	0.004	0.013
LOQ (mM)	0.013	0.045
Repeatability (RSD, %) (*n* = 6)	5.9 (0.02 mM)	3.7 (0.3 mM)
	3.8 (0.05 mM)	3.3 (0.85 mM)
Reproducibility (RSD, %) (*n* = 6)	6.1 (0.02 mM)	5.0 (0.3 mM)
	4.0 (0.05 mM)	3.5 (0.85 mM)
