# Supplementary material for: Colorimetric 3D microPAD for Multiplexed Detection of Paracetamol and Aspirin in Urine and Saliva
Source: Sensors (Basel). 2025 Mar 12;25(6):1756. doi: 10.3390/s25061756 (PMC11945238; doi:10.3390/s25061756)
Supplement: Supplementary file 1 [file sensors-25-01756-s001.zip › sensors-3518841-supplementary.pdf]

Supplementary Material

Colorimetric 3D microPAD for Multiplexed Detection of Paracetamol and Aspirin in Urine and Saliva

Alberto Abalde-Pujales, Vanesa Romero *, Isela Lavilla and Carlos Bendicho *

Centro de Investigación Mariña, Universidade de Vigo, Departamento de Química Analítica y Alimentaria, Grupo QA2, 36310 Vigo, Spain; alberto.abalde.pujales@uvigo.gal (A.A.-P.); isela@uvigo.gal (I.L.)

* Correspondence: bendicho@uvigo.es (C.B.); vromero@uvigo.gal (V.R.); Tel.: +34-986812281 (C.B.)

CONTENTS

S1. Procedure for synthetic saliva and urine samples

Synthetic saliva (1) is made up of the following composition: 100 mL each of 25 mM KH_2PO_4, 150 mM $NaHCO_3$, 100 mM NaCl, and 1.5 mM $MgCl_2$. To this, 6 mL of 25 mM citric acid and 100 mL of 15 mM $CaCl_2$ were added, with the final volume adjusted to 1 liter.

Synthetic saliva (2) is made up of the following composition: 100 mL each of 10.2 mM NaCl, 10.7 mM KCl, 0.29 mM $MgCl_2$, 1.08 mM $CaCl_2$, 4.6 mM K_2HPO_4, and 2.4 mM $NaHCO_3$, with the final volume adjusted to 1 liter.

Synthetic urine (1) contains 100 mL each of 83.25 mM urea, 1.16 mM sodium citrate, 0.22 mM oxalic acid, 7.04 mM Na_2SO_4, 3.6 mM $CaCl_2$, 4.2 mM $MgCl_2$, and 9.35 mM NH_4Cl, with the final volume adjusted to 1 liter.

Synthetic urine (2) contains 100 mL each of 12 mM Na_2SO_4, 1.5 mM uric acid, 2.5 mM sodium citrate, 250 mM urea, 7.8 mM creatinine, 30.9 mM KCl, 31 mM NaCl, 1.7 mM $CaCl_2$, 23.7 mM NH_4Cl, 0.19 mM potassium oxalate, and 4.4 mM $MgSO_4$.
